# Postural and longitudinal variability in seismocardiographic signals

**DOI:** 10.1088/1361-6579/acb30e

**Published:** 2023-02-27

**Authors:** Md Khurshidul Azad, Peshala T Gamage, Rajkumar Dhar, Richard H Sandler, Hansen A Mansy

**Affiliations:** 1 Biomedical Acoustic Research Lab, University of Central Florida, Orlando, FL 32816, United States of America; 2 Biomedical Acoustics Research Company, Orlando, Florida, United States of America; 3 Florida Tech, Melbourne, FL 32901, United States of America

**Keywords:** non-invasive measurements, seismocardiography, cardiac monitoring, postural variability, longitudinal variability

## Abstract

*Objective*. Low frequency cardiovascular vibrations detectable on the chest surface (termed seismocardiography or SCG) may be useful for non-invasive diagnosis and monitoring of various cardiovascular conditions. A potential limitation of using SCG for longitudinal patient monitoring is the existence of intra-subject variability, which can contribute to errors in calculating SCG features. Improved understanding of the contribution of intra-subject variability sources may lead to improved SCG utility. This study aims to quantify postural and longitudinal SCG variability in healthy resting subjects during normal breathing. *Approach*. SCG and ECG signals were longitudinally acquired in 19 healthy subjects at different postures (supine, 45° head up, and sitting) during five recording sessions over five months. SCG cycles were segmented using the ECG R wave. Unsupervised machine learning was used to reduce SCG variability due to respiration by grouping the SCG signals into two clusters with minimized intra-cluster waveform heterogeneity. Several SCG features were assessed at different postures and longitudinally. *Main results*. SCG waveform morphological variability was calculated within each cluster (intra-cluster) and between two clusters (inter-cluster) at each posture and data collection session. The variabilities were significantly different between the supine and sitting but not between supine and 45° postures. For the 45° and sitting postures, the intra-cluster variability was not significantly different, while the inter-cluster variability difference was significant. The energy ratio between different frequency bands to total spectral energy in 0.5–50 Hz were calculated and were comparable for all postures. The combined cardiac timing intervals from the two clusters showed significant variation with postural changes. There was significant heart rate difference between the clusters and between postural positions. The SCG features were compared between longitudinal sessions and all features were not significantly different, *Significance*. Several SCG features significantly varied with posture suggesting that posture needs to be specified when comparing SCG changes over time. Longitudinally comparable SCG feature values suggests that significant longitudinal differences, if observed, may reflect true alternations in the cardiac functioning over time.

## 1. Introduction

Seismocardiographic (SCG) signals are chest surface accelerations induced by cardiac activity such as valve closures, blood momentum changes, and cardiac muscle contraction (Bozhenko, 1961) (Zanetti and Salerno 1990, Crow *et al* 1994, Tavakolian *et al* 2012, Taebi *et al* 2017, Taebi and Mansy 2017a, Taebi, 2018). Several studies suggested that SCG signals contain information that may be used for diagnosing and monitoring heart conditions (Inan *et al* 2015, Taebi *et al* 2019). Potentially useful SCG features include electro-mechanical cardiac time intervals; plus spectral, heart rate and morphological variability (Shafiq *et al* 2016, Yang and Tavassolian 2017, Taebi and Mansy 2017b, Taebi *et al* 2018).

SCG may be used for longitudinal non-invasive monitoring of patient cardiovascular status. SCG signals are, however, known to contain significant intra-subject variability, which may limit the accuracy of extracting clinically useful SCG features (Sandler *et al* 2019). Hence, understanding the sources of intra-subject variability and quantifying their effects may help increase the clinical utility of SCG. Known sources of intra-subject variability include respiration (Azad *et al* 2019, Sandler *et al* 2019), posture (Javaid *et al* 2016a, 2016b), and physical activity levels (Javaid *et al* 2016a, 2016b). An earlier study (Javaid *et al* 2016a, 2016b) reported a variation in the SCG morphology and spectral energy with postural changes (for supine, seated, and standing positions) in heart failure patients. In addition, cardiac timing interval were found to be noticeably different between supine and standing in healthy humans (Di Rienzo *et al* 2013). Another recent study reported that SCG may vary over time even when respiration, postures, and physical activity levels are kept similar, which suggests that there may be additional sources of intra-subject variability (Taebi *et al* 2019).

The objective of this article is to quantify the following intra-subject variabilities:

(a) Postural SCG variability. Here, the supine, 45° tilt, and sitting positions were chosen since these are the more likely postures for acquiring SCG from patients with severe cardiac conditions (with the supine positions being less favorable due to potential increased shortness of breath in that position).
(b) Longitudinal SCG variability. Here, SCG was recorded during sessions spanning a 5 month period. This period was chosen to approximate possible monitoring intervals for patients with severe cardiac conditions.

## 2. Methods

### 2.1. Experimental protocol

After institutional IRB approval, 19 healthy subjects with no known medical history of cardiovascular disease (8 males and 11 females, age: 20–32 years) were recruited for the study. Subject’s demographics are listed in table 1.

**Table 1.** Subjects’ information.

Age (years	23 ± 3.5
Height (cm)	168.6 ± 9
Weight (kg)	69.5 ± 12.7
BMI	24.4 ± 3.7

Subjects were asked to refrain from food and caffeinated drinks as well as avoid heavy exercise for at-least 4 h prior to the study to help exclude potential effects of exercise and food digestion on physiological conditions affecting SCG.

**Figure 1.**
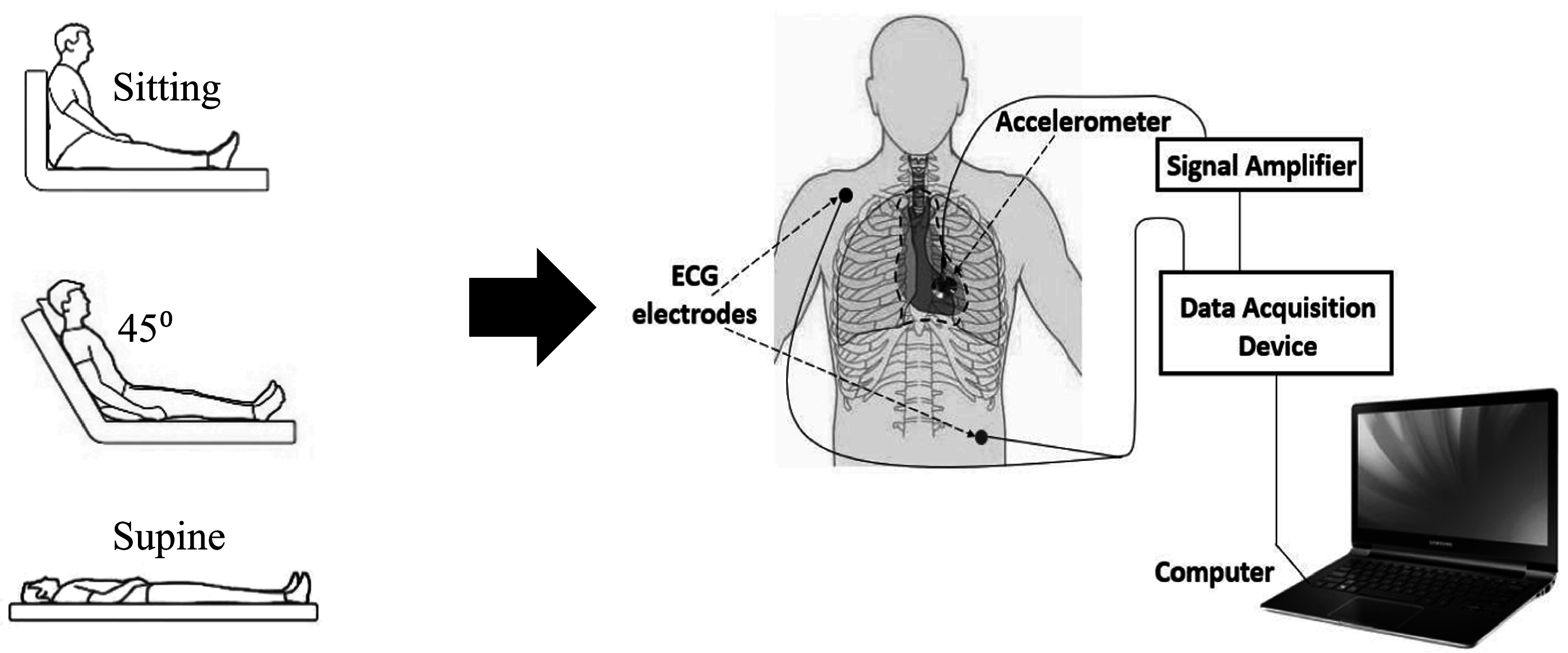
Schematic diagram of the experimental setup, sensor location, and subject postures.

The ECG signal was acquired by a biopotential recorder (IX-B3G, iWorx Systems, Inc., Dover, NH). Seismocardiographic signals were acquired using a tri-axial accelerometer (Model: 356A32, PCB Piezotronics, Depew, NY), which was attached on the chest surface using double-sided medical grade tape at the 4th intercostal space left lower sternal border. The sensor location was chosen according to previous study (Azad *et al* 2021). The accelerometer measured acceleration in the dorsoventral, lateral and craniocaudal directions. The current study emphasizes the dorsoventral-component (i.e. movement normal to the chest wall). The accelerometer signal was amplified using a signal conditioner (Model: 482 C, PCB Piezotronics, Depew, NY) with a gain of 100. The subject was at rest on an exam table for 10 min prior to data collection. Data was collected for 5 min for each of 3 different postures: (a) supine; (b) 45° head up with legs horizontal; and (c) 90° sitting with legs horizontal. A sampling frequency of 10 kHz was used for data acquisition. These measurements were repeated for four more recording sessions over 5 months period. The acquired data was analyzed using MATLAB (Matlab 2018, Mathworks, Natick, MA). The experimental setup is shown in figure 1.

### 2.2. Filtering and SCG segmentation

A previous study (Pandia *et al* 2013) suggested that SCG signal contains significant energy up to 50 Hz for healthy subjects. Hence, SCG and ECG signals were forward-backward filtered using a 4th order Chebyshev 2 type band-pass filter (cut off 0.5–50 Hz) to reduce background noise and baseline wondering due to respiration. In addition, a moving average filter of order 5 was employed to smooth the signal further. The SCG signal was segmented into SCG events (also called heartbeats) using the R wave locations of the ECG signal, which were detected using Pan Tomkins algorithm (Pan and Tompkins 1985). Each SCG event was chosen to start 0.1 s before the R peak of the corresponding ECG and end at 0.1 s before the next R peak. The segmented SCG events were then down sampled to 1000 Hz for faster execution of further analysis.

### 2.3. Unsupervised machine learning to reduce SCG variation with respiration

**Figure 2.**
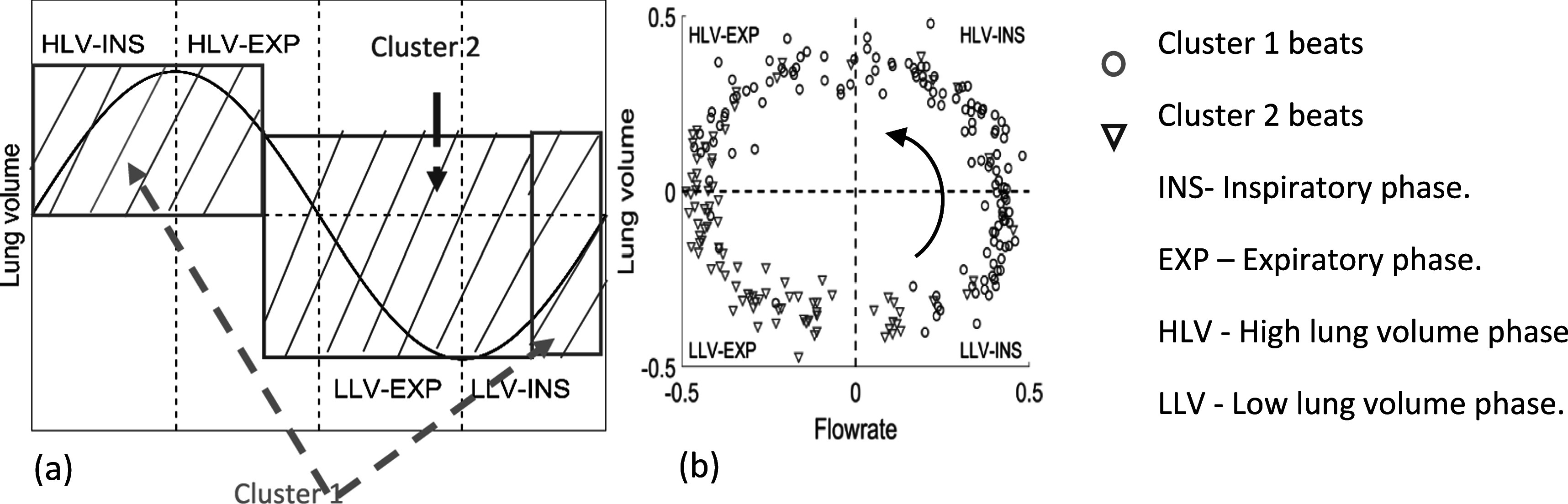
(a) SCG cluster location during a respiratory cycle (as a function of lung volume) after applying unsupervised ML. Cluster 1 SCG events begin at the LLV-INS phase and continue till the HLV-EXP phase. Cluster 2 starts at the HLV-EXP phase and continues till the early LLV-INS phase. (b) SCG cluster assignment in a lung volume and respiratory flow rate space. Blue circles and red triangles show the SCG beats with their respiratory phase suggesting that cluster switching takes place at LLV-INS and HLV-EXP phase.

Precise estimation of SCG features may be impeded by the variation on the SCG with respiration. Previous work in ballistocardiogram waveforms showed significant morphological changes due to changes respiratory phase (Tavakolian *et al* 2008). In addition, seismocardiography studies (Sandler *et al* 2019, Gamage *et al* 2020) illustrated that SCG event morphology have coherent relations with the respiratory phases. Unsupervised machine learning (ML) can optimally cluster SCG beats into two groups which reduces the variation in SCG waveform stemmed from respiration. After clustering, intra- and inter-cluster variability can be calculated. For example, variability of SCG waveforms within an individual cluster (e.g. cluster 1 in figure 2) will be a measure of the intra-cluster variability. On the other hand, SCG waveform variability between clusters (i.e. inter-cluster variability) would provide a measure of waveform variation between, for example, the two clusters shown in figure 2. These variabilities were found useful in predicting heart conditions (Gamage 2020). In the current study, k-medoid clustering was used with dynamic time warping (DTW) as a variability measure to cluster the morphology of SCG events. This clustering method has proven higher accuracies over other methods for shape-based (i.e. morphology-based) clustering of time series (Paparrizos and Gravano 2017). Figure 2 shows an example of the of SCG cluster distribution with respect to respiratory phases.

**Figure 3.**
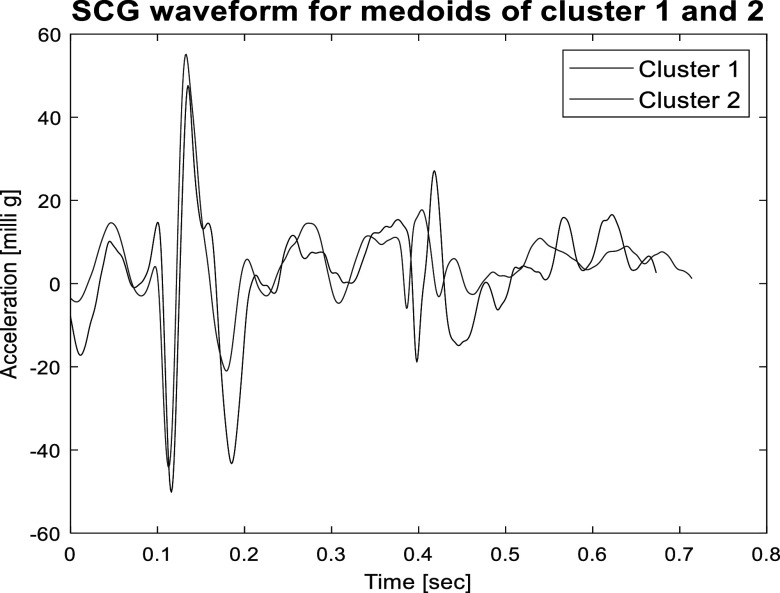
An example of SCG waveform for medoid of cluster 1 and 2. There is noticeable morphological variability between the two cluster medoids due to respiratory variation.

Figure 2 shows that SCG clusters don’t separate entirely on respiratory flow or lung volume phases. In fact, most cluster 1 events occur during late LLV-INS phase to early HLV-EXP phase in the respiratory cycle while cluster 2 events occur from late HLV-EXP phase to early LLV-INS phase. Post clustering, the clustered SCG beats can be represented by the medoid SCG beat (i.e. the median beat) of each cluster (Gamage *et al* 2020) and can be further analyzed to estimate accurate SCG features. Figure 3 shows an example of SCG medoid events of cluster 1 and 2 from a single data recording session.

### 2.4. DTW to calculate morphological variability

**Figure 4.**
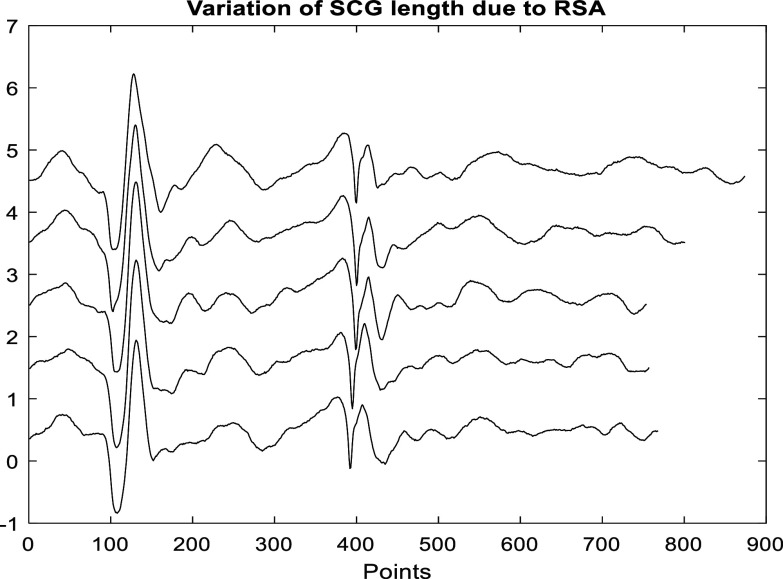
Variation of SCG signal length due to respiratory sinus arrhythmia.

SCG events are known to be nonlinearly stretched due to heart rate variability (e.g. due to respiratory sinus arrythmia (RSA)). These HR variations would lead to significant misalignments between different SCG events, causing discrepancies in the clustering results if Euclidean distance is used as a variability measure. Figure 4 shows the variation in several consecutive SCG event lengths.

**Figure 5.**
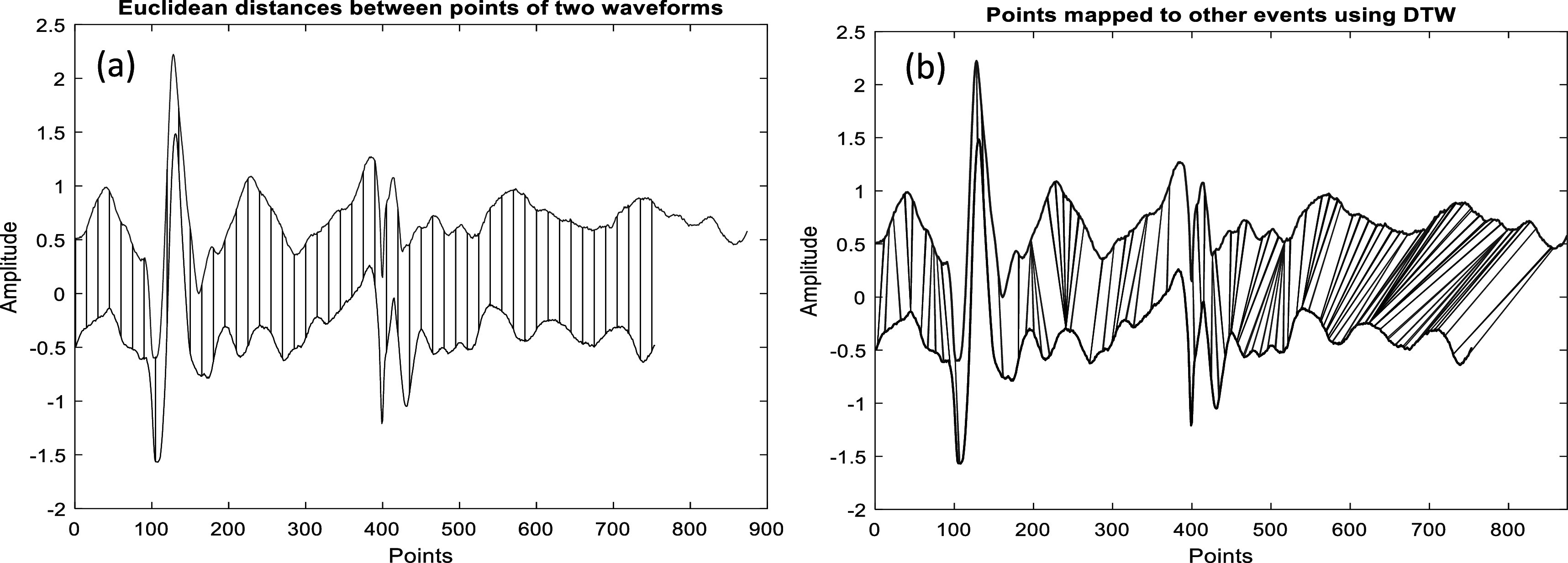
An illustration of distance measure using (a) Euclidean distance (b) DTW distance between two SCG signal. For convenience few points in each signal that corresponds to other signal are shown here.

For precise estimation of morphological variability, the DTW method is used to find the optimal ‘global alignment’ between two-time sequences by exploiting the temporal distortions between them (Sakoe *et al* 1978). A representation of the differences between DTW and Euclidean distance is shown in figure 5.

The clustering algorithm was implemented in MATLAB and is provided below.

Algorithm:

Inputs: number of clusters = *K*. Set of SCG events: $\left\{{X}_{1},\,{X}_{2},{X}_{3},\ldots .{X}_{i}\ldots \,{X}_{n}\right\}$ where each event is defined by its feature vector (amplitude) as ${X}_{i}=\left\{{x}_{1},\,{x}_{2},{x}_{3},\ldots .{x}_{i}\ldots {x}_{n}\right\}.$ *N* is the number of events.

Step 1: initialize ${C}_{1},\,\ldots .,{C}_{j},\,\ldots ..{C}_{k}$ as the medoids.

Step 2: for each ${X}_{i},$ find the nearest ${C}_{j}$ and assign ${X}_{i}$ to cluster j using DTW as the distance measure.

Step 3: update ${C}_{j}$ based on the clustered events from previous step using equation (1).

\begin{eqnarray*}{C}_{j}={{\mathrm{argmin}}}_{y\in \left\{{X}_{1j},\,{X}_{2j},\,\ldots .{X}_{ij}\ldots {X}_{nj}\right\}}\displaystyle \sum _{i=1}^{{n}_{j}}dtw\left(y,{X}_{ij}\right),\end{eqnarray*}

where, *X*_*ij*_ is the *i*th event belongs to cluster *j* and *n*_*j*_ is the number of events that belong to *j* after step 2.

Step 4: repeat step 2 and 3 till none of the cluster assignments change.

The time complexity of DTW is ${\mathrm{\Theta }}\left({l}^{2}\right),$ where *l* is the length of the SCG event (Petitjean *et al* 2014). To reduce the computational time of clustering in the current study, SCG events were down sampled to 1000 Hz. Before clustering, SCG events were also normalized by their maximum amplitudes, which is not expected to affect DTW measure.

### 2.5. SCG features

The analyzed SCG features in the current study include:

1. Morphological variability.
2. Spectral distribution.
3. Cardiac timing intervals.

In addition, the heart rate variability derived from the ECG signal is discussed as it may be useful for predicting cardiac health (Liu *et al* 2014).

#### 2.5.1. Morphological variability

SCG morphological variability was estimated using intra-cluster variability and inter-cluster variability. These SCG features characterize the beat-to-beat SCG variation and may provide useful information about respiratory effects on the SCG signal. To quantify the morphological variability (i.e. the intra and inter-cluster distances), the following equations were used

\begin{eqnarray*}\begin{array}{l}{\mathrm{Intra}}-{\mathrm{cluster}}\,{\mathrm{Distance}}=\,\displaystyle \frac{1}{{n}_{1}+{n}_{2}}\left[\displaystyle \sum _{i=1}^{{n}_{1}}dtw\left({C}_{1},{X}_{i1}\right)+\displaystyle \sum _{i=1}^{{n}_{2}}dtw\left({C}_{2},{X}_{i2}\right)\right]\end{array}\end{eqnarray*}

\begin{eqnarray*}\begin{array}{l}{\mathrm{Inter}}-{\mathrm{cluster}}\,{\mathrm{Distance}}=\,\displaystyle \frac{1}{{n}_{1}+{n}_{2}}\left[\displaystyle \sum _{i=1}^{{n}_{1}}dtw\left({C}_{2},{X}_{i1}\right)+\displaystyle \sum _{i=1}^{{n}_{2}}dtw\left({C}_{1},{X}_{i2}\right)\right].\end{array}\end{eqnarray*}

**Figure 6.**
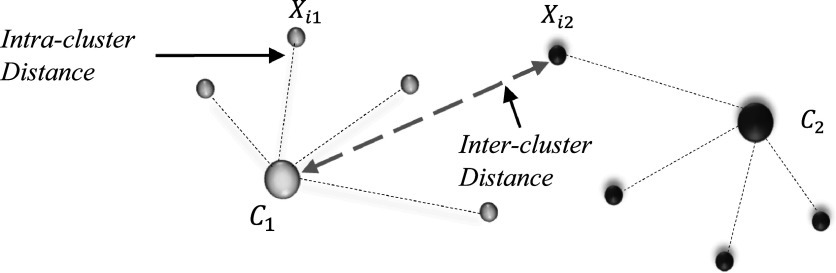
An illustration of intra- and inter-cluster distance (variability). *C*_1_ and *C*_2_ are the cluster medoids of the two clusters. Intra-cluster distance is defined as the distance of *i*th event of cluster 1 *X*_*i*1_ from its own medoid *C*_1_ and inter-cluster distance is the distance of *i*th event of cluster 2 *X*_*i*2_ from the medoid of cluster 1, *C*_1_.

Here, *X*_*i*1_, *X*_*i*2_ are the *i*th SCG event belonging to cluster 1 and 2, respectively while *C*_1_ and *C*_2_ are the respective cluster medoids. And *n*_1_, *n*_2_ are the total number of events belong to groups 1 and 2, respectively. In equations (2) and (3), the function *dtw* is used to quantify the morphological difference between two SCG beats using DTW dissimilarity measure, which was discussed in the previous section. Well separated groups are expected to have relatively low intra-cluster distance and high inter-cluster distance. Figure 6 demonstrates the intra and inter-cluster distance of SCG events from the cluster medoids.

#### 2.5.2. Spectral distribution

**Figure 7.**
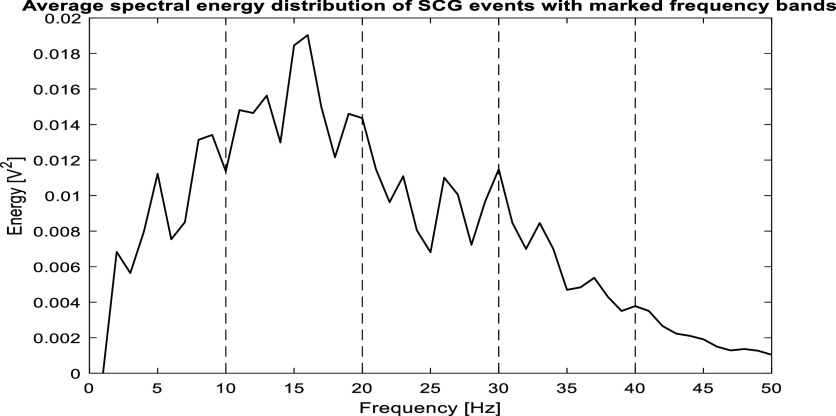
Average spectral energy of SCG events at different frequency bands.

Previous studies (Taebi and Mansy 2017a, Taebi *et al* 2019) suggested that the SCG signal may consist of three dominant frequencies around 9 Hz, 25 Hz, and 50 Hz. The study postulated that the lowest frequency may indicate ventricular contraction while the higher frequencies may correspond to atrioventricular valve closures. SCG’s spectral distribution may correlate with cardiac health. To quantify the SCG spectral distribution, average spectral energy in different frequency bands with respect to the total energy (0.5–50 Hz) was analyzed. Figure 7 illustrates an example of average spectral energy of a group of SCG events in a recording session with frequency bands marked (0.5–10, 11–20, 21–30, 31–40 and 41–50 Hz). Here, the spectral distribution was calculated as the average of the distribution of the 2 cluster medoids. This approach was implemented as suggested in an earlier study (Gamage *et al* 2020).

#### 2.5.3. Cardiac timing interval

**Figure 8.**
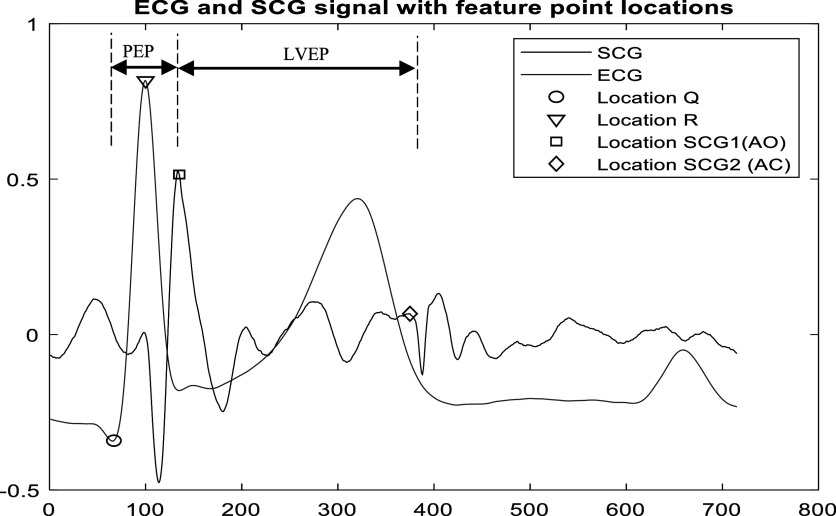
An illustration of pre-ejection period (PEP) and left ventricular ejection period (LVEP) along with ECG and SCG signal.

A previous study (Di Rienzo *et al* 2013) suggested that cardiac timing intervals varied with posture. The current study investigated pre-ejection period (PEP) and left ventricle ejection period (LVEP) variation with respect to posture. SCG feature points were identified according to previous study (Crow *et al* 1994). Here, PEP was estimated from *Q* point of ECG wave to SCG1 peak, which correlates with aortic opening (AO) while LVEP was measured from SCG1(AO peak) to SCG2, which correlates with aortic closure (AC peak). Both PEP and LVEP were estimated for the 2 cluster medoids and their average was calculated. Figure 8 shows how PEP and LVEP can be identified using simultaneously recorded SCG and ECG signals.

#### 2.5.4. Statistical analysis

SCG features were compared between the postures (for postural variability) and among the recording sessions (for longitudinal variability). To compare the difference of means between two groups (e.g. the SCG features for two postures), pairwise students *t*-test was performed using following equation

\begin{eqnarray*}t=\displaystyle \frac{\mu \,* \,\sqrt{n}}{\sigma }.\end{eqnarray*}

Here, *μ* is the average of the paired differences, *σ* is the standard deviation of paired differences and *n* is the number of samples. The *p*-value was then found from the *t*-table. To compare the ‘effect size’ of the differences between the two groups (e.g. the SCG features for two postures), ‘Cohen’s *d* (Cohen 1988) was calculated using the following equations:

\begin{eqnarray*}d=\frac{{\mu }_{1}-{\mu }_{2}}{{\sigma }_{\mathrm{pooled}}}\end{eqnarray*}

\begin{eqnarray*}{\sigma }_{\mathrm{pooled}}=\sqrt{\frac{\left({n}_{1}-1\right){\sigma }_{1}^{2}+\left({n}_{2}-1\right){\sigma }_{2}^{2}}{\left({n}_{1}+{n}_{2}-2\right)}}.\end{eqnarray*}

Here *μ*_1_ and *μ*_2_ are the group averages and ${\sigma }_{\mathrm{pooled}}$ is the pooled standard deviation. ${\sigma }_{1},$ ${\sigma }_{2}$ are standard deviation of group 1 and 2 with sample size of ${n}_{1}$ and ${n}_{2}$ respectively. The Cohen’s *d* is considered small, medium, large and very large when *d* < 0.2, 0.2 < *d* < 0.5, 0.5 < *d* < 0.8, and 0.8 < *d*, respectively.

To compare SCG features over five recording sessions longitudinally one-way analysis of variance (ANOVA) was used using the following equation

\begin{eqnarray*}F-\mathrm{statistic}=\frac{\mathrm{variation}\,\mathrm{between}\,\mathrm{groups}\,}{\mathrm{variation}\,\mathrm{within}\,\mathrm{groups}\,}.\end{eqnarray*}

The *p*-value corresponding to the *F*-statistic value was obtained from the *F* distribution table.

## 3. Results and discussion

To document the effect of posture on SCG features, the features under consideration were compared among postures. The effect size was then calculated along with the statistical significance of feature changes between postural positions, which may help guide optimal postural position choices. Furthermore, the variations of these features were evaluated over five recording sessions at different intervals to estimate the longitudinal variations of these feature. This would help find optimal (i.e. longitudinally stable) features that may be used for monitoring patients with cardiac conditions over long periods of time.

### 3.1. Postural effects

#### 3.1.1. Morphological waveform variability

**Figure 9.**
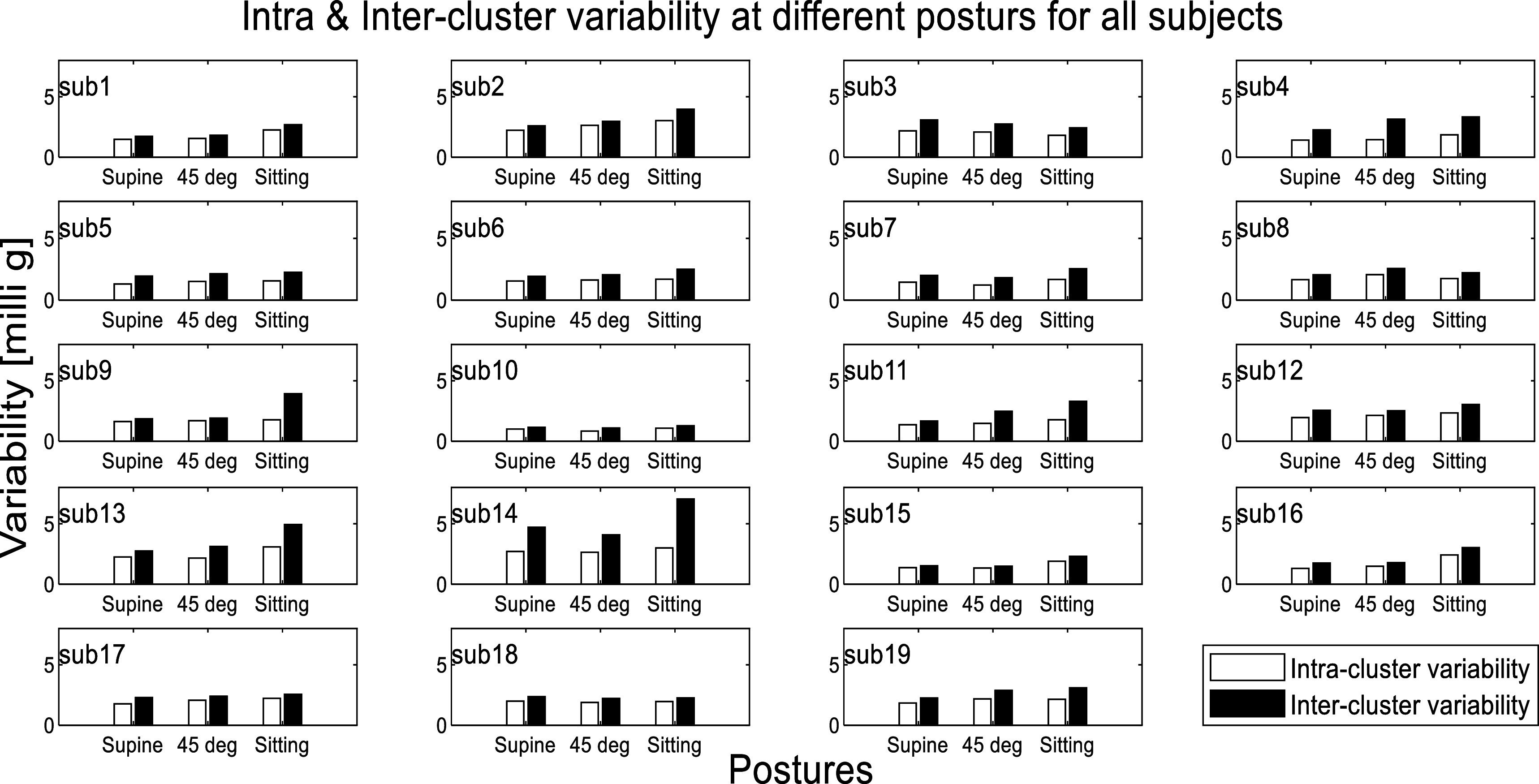
Intra and inter-cluster variability in all subjects. The intra-cluster variability values were lower than inter-cluster variability suggesting appropriate clustering.

The intra and inter-cluster SCG morphological respiratory variabilities (described in section II.E) may provide useful features that are predictive of cardiac health (Sandler *et al* 2019). The intra and inter-cluster variability at different postures for all subjects is plotted in figure 9.

**Figure 10.**
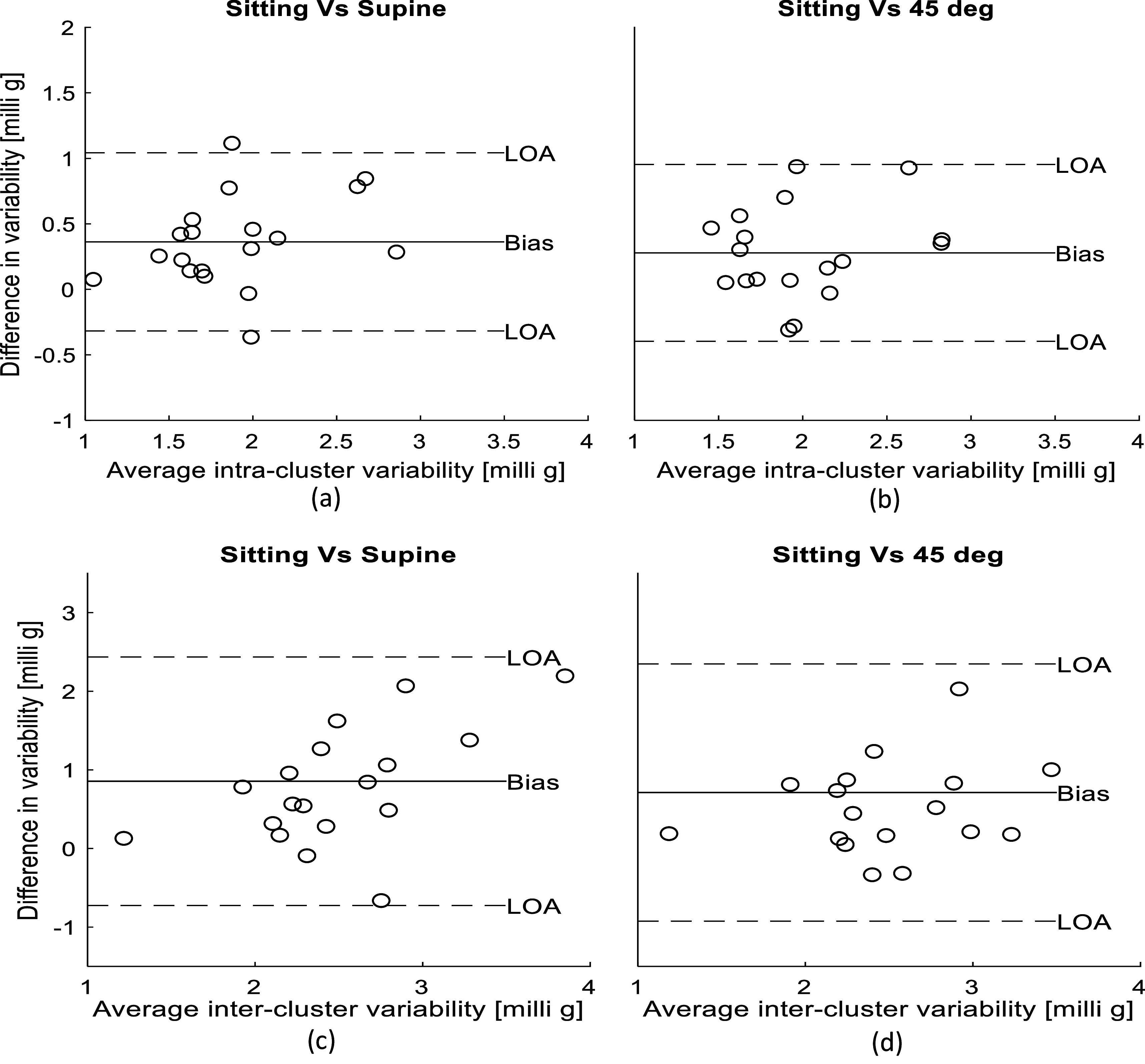
Bland–Altman analysis of intra-cluster variability between (a) sitting and supine (b) sitting and 45° tilt. Inter-cluster variability between (c) sitting and supine (d) sitting and 45° tilt. The bias was positive in all cases, suggesting that the variability is lower in the supine and 45° relative to sitting.

Figure 9 suggests that the intra-cluster variability was smaller than inter-cluster variability in all the subjects, as expected. This indicates an appropriate separation between SCG clusters, which is consistent with a previous study (Azad *et al* 2019). The intra-cluster variability ranges from 1 to 2 milli g while inter-cluster variability ranges from 2 to 5 milli g for most subjects. To compare the intra and inter-cluster variability between postures, Bland–Altman (Bland and Altman 1999) plots are shown in figure 10.

Figure 10 suggests that both intra and inter-cluster variability tend to be higher in sitting position compared to supine and 45°. To investigate the significance and effect size of this variability between the postural position, the pairwise *t*-test along with ‘Cohen’s *d* effect size were calculated and shown in table 2.

**Table 2.** Pairwise *t*-test and ‘Cohen’s *d* effect size for changes in intra and inter-cluster variability between postures for the study subjects.

Paired test and effect size for		Intra-cluster variability		Inter-cluster variability	
Postures		*P* value (2-tailed)	Cohen’s *d* effect size	*P* value (2-tailed)	Cohen’s *d* effect size
Pair 1	Supine—45°	0.536	0.10	0.694	0.06
Pair 2	45°—sitting	0.074	0.41	0.043	0.59
Pair 3	Supine—sitting	0.005	0.57	0.005	0.73

Table 2 suggests that there is no significant difference in the intra-cluster and inter-cluster variability values between supine and 45° for both intra and inter-cluster variability with a small effect (‘Cohen’s *d* < 0.5), while significant variability differences exist with medium effect (0.5 < Cohen’s *d* < 0.8) between supine and sitting. The intra cluster variability difference was found to be not significant between 45° and sitting, while the inter cluster variability difference was significant with a medium effect. This data also suggests that SCG morphology at 45° had more similar intra and inter-cluster variation relative to the supine than the sitting position. It may then be concluded that in a clinical setting, SCG acquired in supine, or 45° posture may result in comparable variability.

#### 3.1.2. Spectral distribution

**Figure 11.**
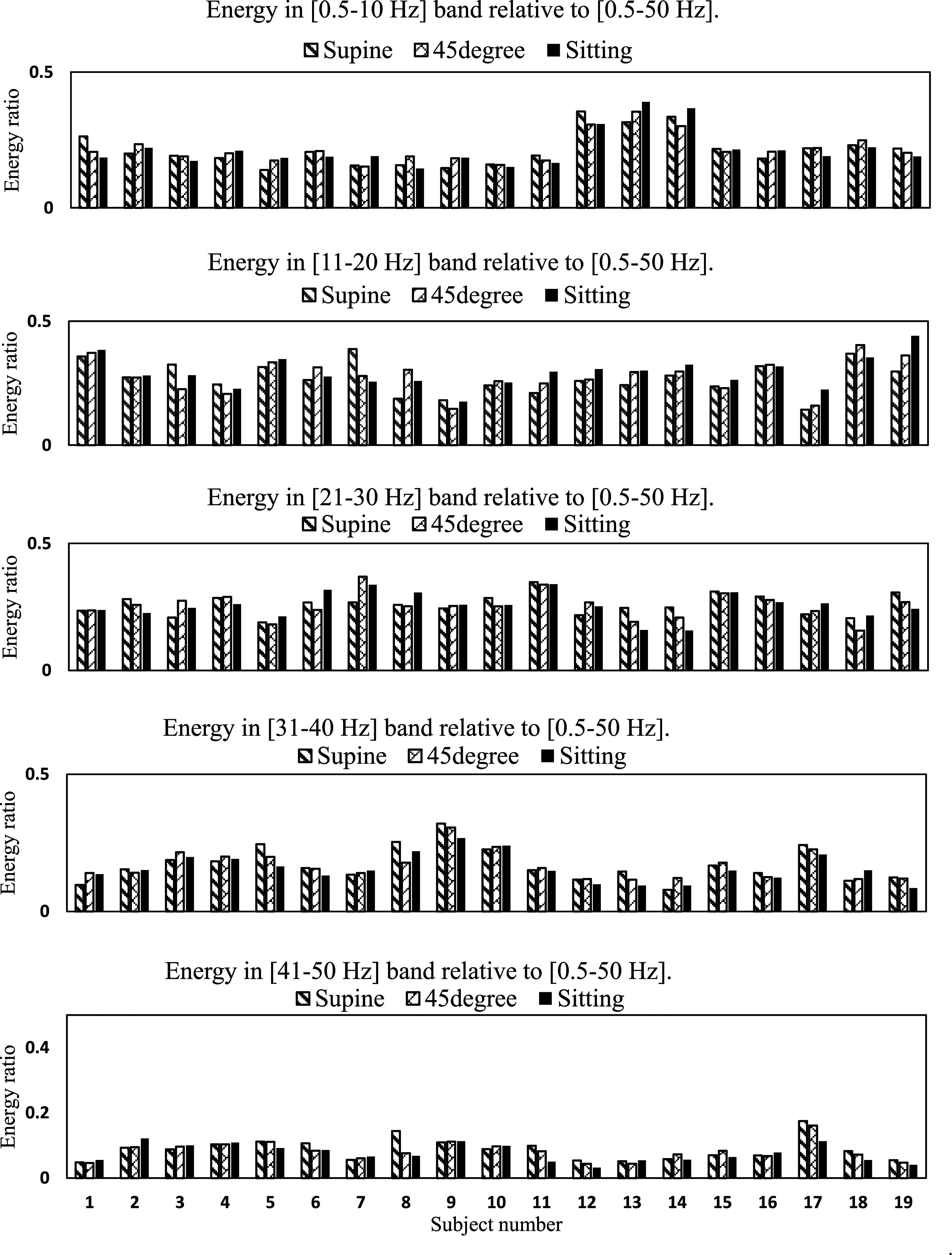
Energy in different frequency bands relative to [0.5–50 Hz]. Energy in [11–20 Hz] tends to be maximum in most subjects.

As mentioned in section II.E, SCG spectral distribution may provide important information about the myocardial movements and valve closure and can be a useful feature in predicting cardiac health. To investigate spectral variations with postural changes, spectral energy at different frequency bands relative to total spectral energy from 0.5 to 50 Hz were studied. Figure 11 showed the spectral energy ratio with respect to total energy from 0.5 to 50 Hz at different frequency bands for 3 different postures and all subjects.

Figure 11 suggest that the maximum SCG energy was in the 11–20 Hz frequency band in most subjects. This spectral distribution also suggests that significant SCG energy tended to be below audible range (<20 Hz). There was no significant difference (*p* > 0.05) in energy ratio between postures. The energy ratio in the 11–20 Hz frequency band was approximately 11% and 15% higher at the sitting position compared to the 45° and supine positions, respectively.

Table 3 showed the pairwise test of energy ratio at different frequency bands for postures.

**Table 3.** Paired samples *t*-test for different frequency bands relative to [0.5–50 Hz] between the 3 postures.

Paired samples *t*-test for different frequency bands relative to [0.5–50 Hz]						
Pair	Postures	*P* value for [0.5–10 Hz]	*P* value for [11–20 Hz]	*P* value for [21–30 Hz]	*P* value for [31–40 Hz]	*P* value for [41–50 Hz]
Pair 1	Supine—45°	0.687	0.482	0.705	0.740	0.187
Pair 2	Supine—sitting	0.815	0.088	0.859	0.102	0.088
Pair 3	45°—sitting	0.914	0.087	0.852	0.053	0.179

Table 3 suggests that energy in different frequency bands between postures are not significantly different (*p* > 0.05). This indicates that the SCG’s frequency domain features (which may be indicative of myocardial movements and valve closures) remain comparable between postures.

#### 3.1.3. Cardiac timing intervals

**Figure 12.**
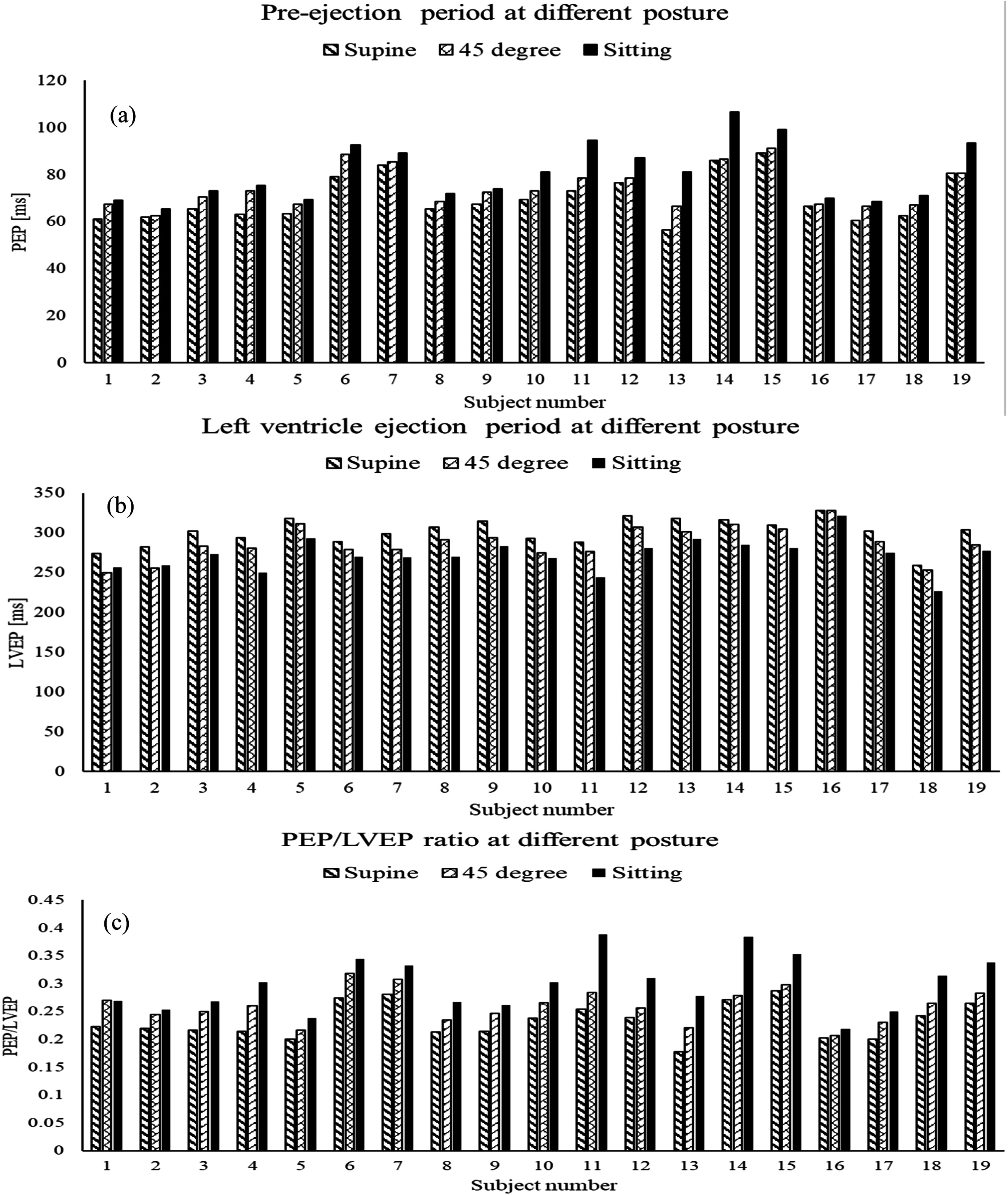
Cardiac timing parameter (a) PEP, (b) LVEP and (c) PEP to LVEP ratio.

As previously discussed, cardiac timing intervals (CTIs) may offer useful information for predicting cardiac health. The cardiac timing interval parameters (PEP, LVEP and PEP to LVEP ratio) with postures are shown in figure 12.

Figure 12 showed that PEP tended to increase as the subject posture varied from supine to sitting (Supine: 70 ± 9.6 ms; 45°: 74 ± 8.6 ms; Sitting: 80.6 ± 12.2 ms) while the LVEP decreased (Supine: 300 ± 17.7 ms; 45°: 286.9 ± 20.9 ms; Sitting: 272 ± 20.4 ms) in most subjects. This variation in cardiac timing followed a similar trend reported in a previous study (Di Rienzo *et al* 2013) that discussed CTI between supine and standing posture. To evaluate the CTI variation between postures, paired sample *t*-test and Cohen’s d effect size for PEP and LVEP are shown in table 4 for posture pairs.

**Table 4.** Paired sample *t*-test and Cohen’s *d* effect size for PEP and LVEP between postures.

Paired sample *t*-test for		PEP		LVEP	
Pair	Posture	*P* value (2-tailed)	Cohen’s *d* effect size	*P* value (2-tailed)	Cohen’s *d* effect size
Pair 1	Supine—45°	1.75E-05	0.46	1.02E-07	0.73
Pair 2	45°—sitting	1.10E-04	0.61	1.63E-05	0.72
Pair 3	Supine—sitting	4.26E-07	0.97	5.75E-11	1.51

Both PEP and LVEP values varied significantly (*p* < 0.05) between postures with medium to large effect (Cohen’s *d* > 0.4). Previous studies (Watanabe *et al* 2007, Di Rienzo *et al* 2013) indicated that the cardiac timing interval changes with respect to posture may be linked to the changes in vagal and sympathetic activity. In the current study, PEP at supine was approximately 6% lower than 45° and approximately 11% lower than sitting position. On the other hand, LVEP was 4% higher at supine with respect to 45° and about 9% higher than that of sitting position. Considering the significant variation in CTIs relative to postural changes, it is imperative for subjects to maintain the same posture when studied over longitudinally.

#### 3.1.4. Heart rate

**Figure 13.**
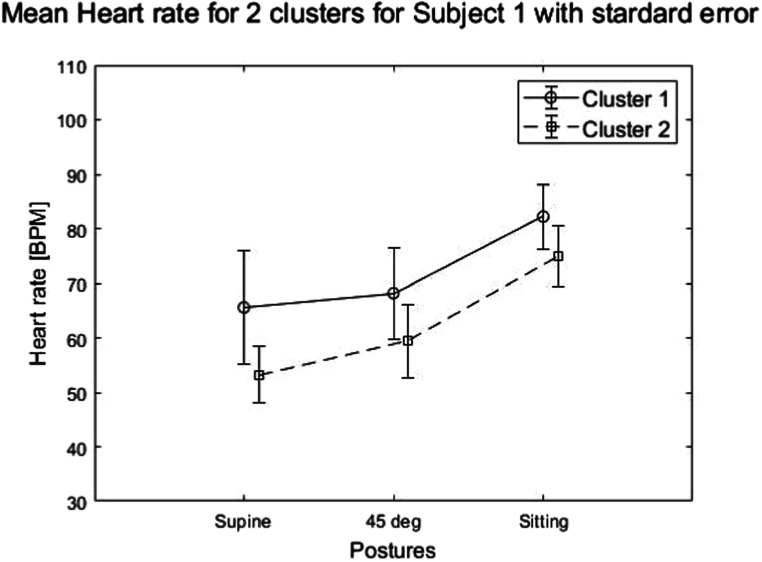
Mean heart rate with standard error in cluster 1 (continuous line) and cluster 2 (dotted line) are shown for a representative subject. Cluster 1 heart rate tend to be higher than Cluster 2. All other subjects showed similar trend.

Heart rate is known to vary with respiratory phases (Hirsch and Bishop 1981, Berntson *et al* 1993, Taebi *et al* 2018). Since the cluster assignments was found to correlate with respiratory phases (Azad *et al* 2019, Gamage *et al* 2020), the corresponding heart rate may also vary between clusters, which may also be of diagnostic value for predicting cardiac health. The current study investigated the heart rate variation between clusters at different postures. Figure 13 showed the mean heart rate and standard error of the two clusters for different postures.

The heart rate between cluster 1 and 2 was found to be significantly different (*P* < 0.05) in 18 of 19 subjects with an effect size *d* = 1.22 (Cohen’s *d* > 0.8) which indicates a large effect in heart rates between clusters.

**Figure 14.**
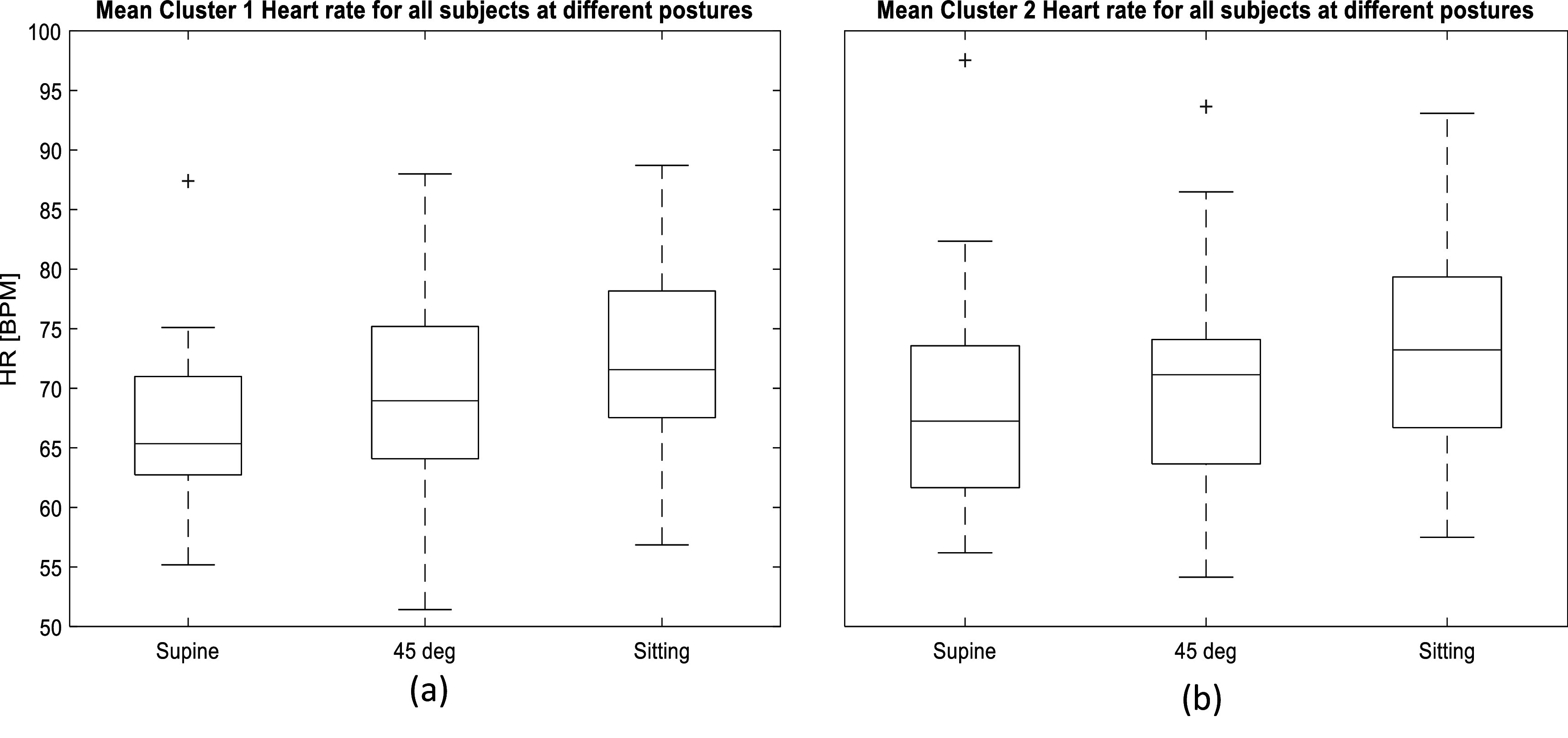
Mean Heart rate for Cluster 1 (a) and Cluster 2 (b). Average heart rate tends to be increased as the postures varied from supine to sitting posture Note that the heart rate differences between the two clusters is small and cannot be seen clearly in this figure. However, a consistent trend in the study subjects was seen similar to figure 13.

Figure 13 suggests that the heart rate for cluster 1 was higher than that of cluster 2 (Gamage *et al* 2020). In addition, the heart rate tended to increase as the posture varied from supine to sitting. The heart rate at sitting was found to be approximately 3% higher than at 45° and approximately 8% higher than supine. Figure 14 shows the average cluster 1 and 2 heart rate for all subjects.

Figure 14 suggests that the average cluster 1 and 2 heart rate for all subjects tend to increase significantly (*P* < 0.05) as the subject varied postures from supine to sitting. The heart rate between clusters was found to be significantly different in most subjects. According to previous studies (Gamage *et al* 2018, Taebi *et al* 2018) this variation of heart rate may due to the change in respiratory phase and corresponding pressure around the heart or possibly due to the autonomic regulation of cardiovascular function (Šipinková *et al* 1997, Watanabe *et al* 2007). This suggests that cardiac activity is affected by postural change, which suggests that in a longitudinal study, a consistent posture would be beneficial for extracting robust features for predicting cardiac health.

### 3.2. Longitudinal variability

**Figure 15.**
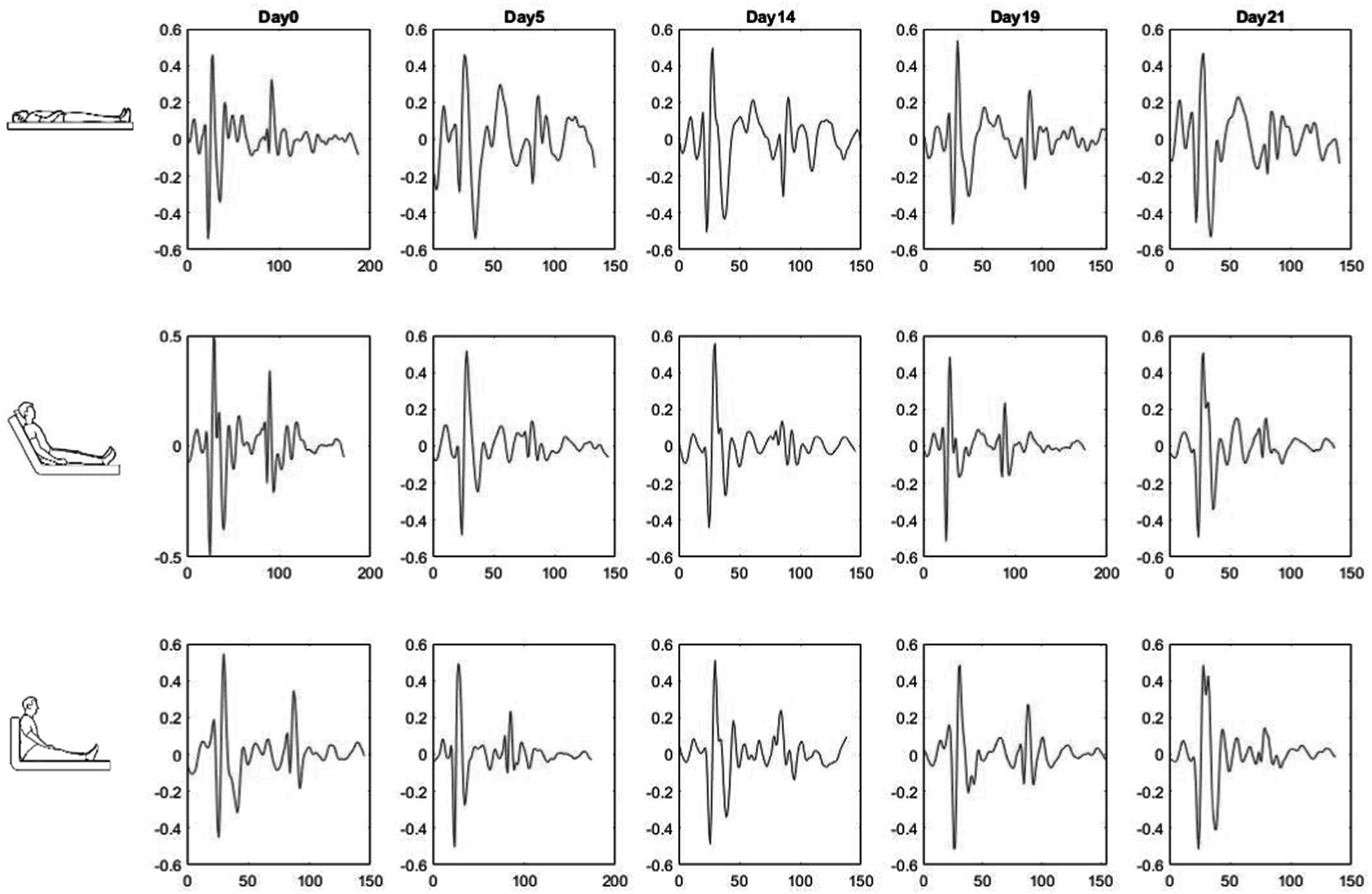
Longitudinal SCG beat variation in a representative subject at different postures.

When monitoring cardiac conditions over time, longitudinal measurements of relevant parameters, including SCG, may be performed. Longitudinal changes in the SCG features can occur with clinical status variations but may also occur due to unknown factors (other than patient status, posture, and physical activity). Figure 15 shows the longitudinal SCG beat variation of a representative subject at different postures during five recording sessions.

This part of the study aims at documenting the longitudinal SCG variability while keeping the subject clinical status, posture, and physical activity level similar. This will help quantify the longitudinal stability of SCG features, which will be key in assessing their utility for longitudinal monitoring of cardiac conditions.

#### 3.2.1. Morphological variability

**Figure 16.**
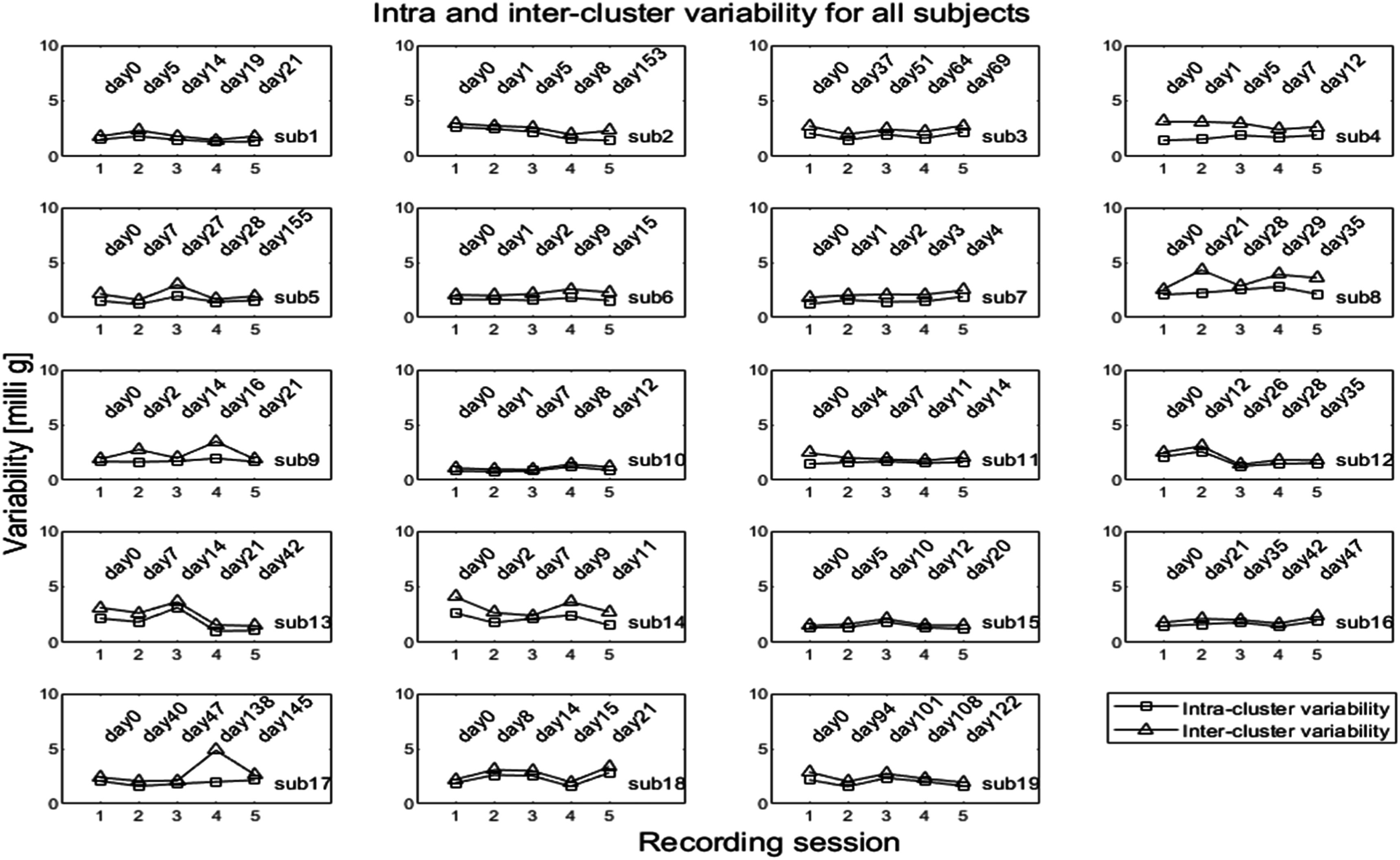
Intra and inter-cluster variability plotted over 5 recording sessions. The intra and inter-cluster variability remained comparable for most subjects over the five sessions.

Figure 16 shows the intra and inter-cluster variability (equations (2) and (3)) of all subjects over 5 different recording sessions.

Figure 16 suggests that the intra-cluster variability varied between 1 and 2.5 milli g while inter-cluster variability varied between 1.5 and 4 milli g for most subjects. It is to be noted that the analysis in this section compared data at the same posture and cluster. Therefore, the effects of posture and respiration are expected to be relatively small, and the changes detected here may be due to other factors that may have changed among sessions. These may include sensor positioning error and other unknown sources. ANOVA was performed for intra and inter-cluster variability to investigate the statistical significance among recording sessions using data for all 19 subjects. The corresponding *p*-values are listed in table 5.

**Table 5.** One-way ANOVA test to compare inter-session variability among all the recording sessions for all the postures and subjects.

ANOVA for longitudinal variation	Intra-cluster variability	Inter-cluster variability
Posture	*p* value	*p* value
Supine	0.49	0.63
45°	0.55	0.99
Sitting	0.51	0.70

Table 5 suggests that the morphological variability (intra and inter-cluster variability) values between recording sessions were not significantly different for the study subjects. This suggest that significant changes in this feature may be utilized to predict changes in cardiac activity, which makes it a potentially valuable feature for potentially monitoring cardiac condition.

#### 3.2.2. Spectral distribution

**Figure 17.**
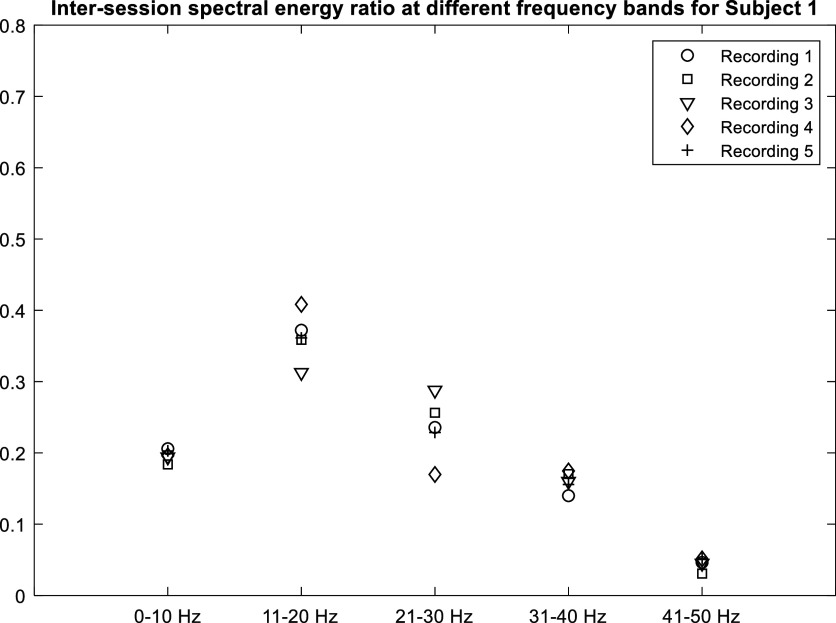
Spectral energy ratio at different frequency band to total energy from 0.05 to 50 Hz over 5 recording session for Subject 1. Spectral energy remained comparable over recording sessions. All other subjects showed similar trend.

The spectral distribution in the 0.05 to 50 Hz band for a representative subject is shown in figure 17. This distribution followed similar trends and was not significantly different (see table 6) among recording sessions. This indicates that the spectral distribution can be a robust feature to utilize for predicting cardiac health. Hence, a significant deviation in features above the levels reported here would indicate potential change in the patient cardiac condition.

**Table 6.** One-way ANOVA test comparing spectral distribution at different frequency bands among all the recording sessions and subjects.

ANOVA for different frequency bands relative to [0.5–50 Hz]					
Posture	*P* value for [0.5–10 Hz]	*P* value for [11–20 Hz]	*P* value for [21–30 Hz]	*P* value for [31–40 Hz]	*P* value for [41–50 Hz]
Supine	0.78	0.85	0.85	0.99	0.59
45°	0.90	0.88	0.90	0.89	0.53
Sitting	0.88	0.89	0.64	0.85	0.74

#### 3.2.3. Cardiac timing intervals

**Figure 18.**
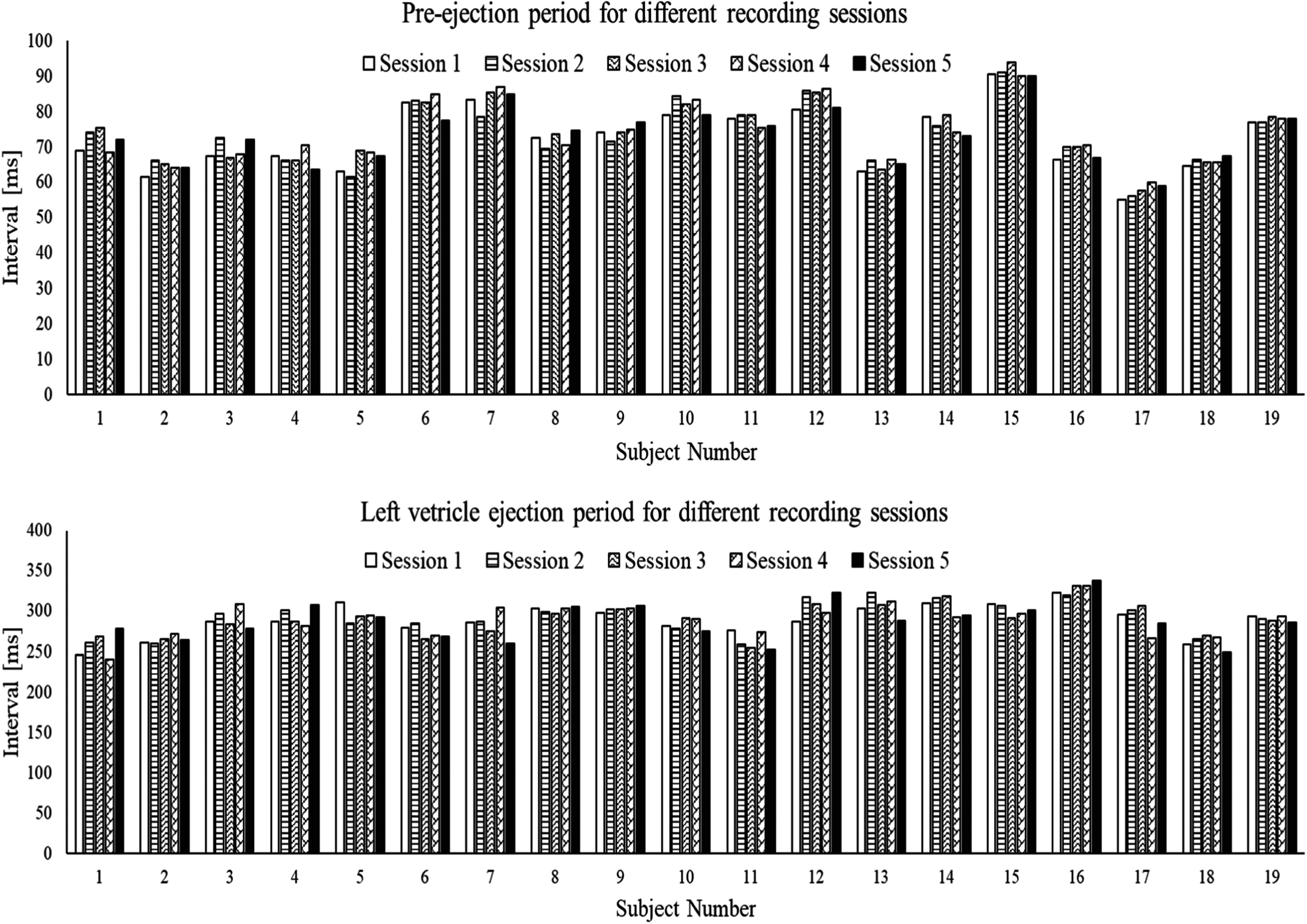
Cardiac timing interval for different recording sessions (a) PEP (b) LVEP for all subjects. Both intervals tend to be comparable among recording sessions.

The cardiac timing interval parameters (PEP, LVEP) is shown in figure 18 for all subjects and recording sessions.

Figure 18 suggests that the cardiac timing intervals remained comparable among different recording sessions, where PEP values varied approximately 3%–4% while LVEP varied 3%–5% (relative to recording session 1). ANOVA was then performed to quantify the statistical significance of inter-session PEP and LVEP changes and results are shown in table 7.

**Table 7.** One-way ANOVA test for PEP and LVEP inter-session changes.

ANOVA for	PEP	LVEP
Posture	*P* value	*P* value
Supine	0.88	0.90
45°	0.96	0.96
Sitting	0.95	0.99

Table 7 suggests that the cardiac timing intervals were not significantly different among recording sessions. This suggests that PEP and LVEP can be useful features for evaluating cardiac activity.

#### 3.2.4. Heart rate

**Figure 19.**
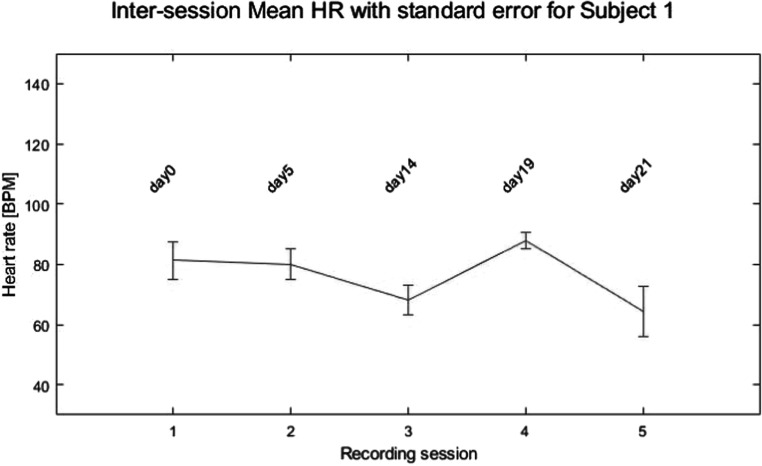
Mean Heart rate with standard error over the 5 recording sessions for a representative subject.

To investigate heart rate variation with respect to recording sessions, the mean heart rate with standard error corresponding to each session was plotted for a representative subject in figure 19.

Figure 19 suggests that the mean heart rate remained similar among the recording sessions. ANOVA was performed to quantify the heart rate changes among session pairs for all subjects and results are shown in table 8.

**Table 8.** One-way ANOVA test to compare mean heart rate among recordings.

ANOVA for longitudinal HR variation	
Posture	*P* value
Supine	0.97
45°	0.88
Sitting	0.80

Table 8 suggests that the mean heart rate did not vary significantly between recording sessions. This further suggests that under similar physiological condition, the heart rate was not significantly different. This would indicate that significant variation in heart rate may be a result of cardiac activity changes. This also gives confidence that the physical activity level among sessions were appropriately controlled.

One of the limitations of this study was the narrow age group of the participating subjects. Future studies need to include subjects with different age groups, especially elderly population who are more susceptible to cardiac diseases to further generalize the study results. The sample size of the study was small and only included healthy subjects. Investigating a larger number of subjects which also included patients with different cardiovascular diseases for a longer period of time is recommended for future studies.

## 4. Conclusion

In this study postural and longitudinal SCG signal variability was studied in 19 healthy subjects. Data was collected at rest for three postures (supine, 45° head up and sitting positions) over about 5 month period. Results suggested that several SCG features (morphological variability, cardiac time intervals) as well as heart rate significantly varied with posture while SCG spectral distribution did not significantly vary. This indicates the need to avoid postural changes when comparing SCG changes over time and among different SCG studies (even if recordings were done at rest). The SCG feature values and heart rate remained comparable over several days (when posture was fixed). The current study focused on the SCG signal features in dorsoventral direction in healthy subjects. Future studies may include SCG signal analysis in the craniocaudal and lateral directions and in patients with cardiac conditions. Future studies may also quantify the SCG signal variability over longer periods. Results of the current study may help establish a baseline for SCG’s postural and longitudinal variability. Feature changes above the observed levels may be suggestive of varying cardiovascular status, including pathologic changes.

## Ethical statement

The study is approved by Institutional Review Board (IRB ID: MOD00001047).
